# A demographic survey of unwanted horses in Ireland in 2011 and totals for 2012 and a comparison with 2010

**DOI:** 10.1186/2046-0481-66-20

**Published:** 2013-10-25

**Authors:** Desmond P Leadon, Rebecca Jeffery, Dylan O’Toole, Vivienne Duggan

**Affiliations:** 1 Irish Equine Centre, Johnstown, Naas, Ireland; 2 Co Kildare & School of Veterinary Medicine, University College Dublin, Belfield, Dublin, Ireland

## Abstract

This report compiles the available information on unwanted horses in Ireland for 2011 and 2012 and builds upon the previous report for the period 2005 to 2010. Similar trends are present in the high value responsible ownership category and the practicing veterinary profession although extensively involved in horse welfare, euthanises a small proportion of Ireland’s unwanted horses. Welfare groups have limited resources and a limited ability to deal with such an extensive problem, which has involved very large numbers of horses. Local authorities continue to have to devote significant efforts and calls on public finances to deal with unwanted horses. Those that they have to deal with are, in the main, not identifiable by either passports or microchips. Category 2 plants and abattoirs continue to provide the principal means of disposal of unwanted horses. The need for abattoirs continues to increase and it is essential that these facilities remain in operation. They processed more than 49,000 horses between 2010 and 2012. The samples they have to submit for Trichinella testing are the most sensitive indicator of the extent of the unwanted horse problem and the most immediate source of information on when it may begin to abate. Trichinella sample numbers and this by inference, horses ponies and donkeys sent to slaughter have fallen by some 35% from 2012 numbers, in the year to date (2013). This may reflect the commercial decision to cease horse slaughter by two slaughterhouses that had hitherto provided this service. Their commercial decision was not in any way related to the identification of fraudulent mislabeled beef in other plants.

## Background

Unwanted horses are a continuing cause of national and international concern [1,2]). In their report on unwanted horses [1,3], stated that there is a need for annual demographic data compilation and review of the numbers of unwanted horses and ponies within the Irish horse industry to assist policy makers and legislators. This report provides an update on the 2005 to 2010 data for the year 2011 and provides government statistics for both 2011 and 2012.

### Aims

This survey aimed to quantify the numbers of horses born, sold, retired, re-homed, euthanised, or sent to Category 2 plants or abattoirs by horse owners in Ireland in the year 2011 and compare this data with that previously published for the years from 2005–2010. It aimed to describe the veterinary involvement in euthanasia and calls to the profession to visit unwanted horses at the request of welfare groups and local authorities. It also aimed to document the numbers of calls, group size, visits, horses actually present and numbers found dead or moribund by welfare groups and Local Authorities and numbers of horses seized, released and sent for slaughter. Central Government data was sought on national involvement in unwanted horses by Local Authorities and the number of horses sent for entry into the human food chain through licensed abattoirs.

The study sought independent corroboration of the central data.

As before, the study framework aimed to provide a template to facilitate an annual review of the demographics and magnitude of the unwanted horse population for industry stakeholders, the veterinary profession, welfare groups, local authorities and national Government to assist with policy making and if required, legislation.

## Methods

### Surveys

A link to an online survey facility (Survey Monkey) was circulated to horse owners, private veterinary practitioners, equine welfare organisations, local authority veterinarians, managers of Category 2 plants and managers of abattoirs slaughtering horses for human consumption.

1,100 members of the Irish Thoroughbred Breeders’ Association (ITBA) were sent the survey by e-mail through the offices of the ITBA and Horse Sport Ireland (HSI) also sent the same survey to the affiliate secretaries of it’s 19 constituent organisations who have a combined total of 20,364 members. 1,222 private veterinary practitioners that were members of Veterinary Ireland (the national representative body for veterinarians) and a further 873 non-member veterinary practitioners received an e-mail survey. A list of equine welfare organisations and sanctuaries was compiled through a web based search and e-mail addresses sought. The survey was circulated online to these organisations. A survey was circulated online to 44 Local Authority Chief Veterinary Officers through Veterinary Ireland. A list of Category 2 plants (38) and abattoirs (five in total – B & F Meats in Kilkenny, Ballon Meats in Co. Carlow, Shannonside Foods in Co. Kildare, Ashgrove Wholesale Ltd in Co Limerick and Ossory Meats in Co. Offaly) was compiled. Surveys were circulated online where e-mail addresses were available. Otherwise, the survey was sent in paper form. The surveys were circulated with a cover letter explaining the purpose of the survey. A reminder was sent after 14 days and the surveys were closed 28 days after circulation.

All questions in the on-line survey related to the year 2011. Horse owners were asked how many equines they kept, sold (by public auction, private sale or to a dealer), retired, re-homed, had euthanised or disposed of via Category 2 plants or abattoirs. They were asked for age (<5, 5–10, 10–15,15-20 and >20 years), breed / type (pony, horse, no breed, sporthorse, thoroughbred) and gender of equines. Members of the practicing veterinary profession were asked how many equines they euthanised for medical conditions or at the owner’s request, or at the request of an Garda Siochana or the local authority. They were also asked how many visits they made to abandoned or neglected equines and how many equines were involved. They were asked for age (<5, 5–10, 10–15,15-20 and >20 years), and breed / type (Pony, Horse, No breed, Sporthorse, Thoroughbred) and gender of equines Welfare groups and local authority veterinarians were asked how many calls they received, and how many visits they made in response to these calls and how many equines were involved in each call or visit. They were asked for age, (<5, 5–10, 10–15,15-20 and >20 years) breed / type (pony, horse, no breed, sporthorse, thoroughbred) and gender of equines they received calls about and they visited. They were asked how many equines they found dead on premises, and how many required immediate or subsequent euthanasia.

Site visits were made to five Welfare societies who were asked in the course of these visits about their acreage available for grazing, staff levels and number of calls they received, those kept on site and fostered, and the principal conditions identified on admission.

Local authorities were asked how many equines they seized, released and euthanised. They were asked for age (<5, 5–10, 10–15, 15–20 and >20 years), breed / type (pony, horse, no breed, sporthorse, thoroughbred) and gender of equines. Managers of Category 2 plants and abattoirs were asked about the throughput of equines in each year and their type and age distribution. National statistics on local authority involvement with unwanted equines and Category 2 plants and abattoirs was obtained from the Department of Agriculture, Fisheries and Food (DAFF).

Data on mandatory Trichinella testing of equine carcasses from Irish abattoirs was obtained from the Microbiology Unit of the Irish Equine Centre.

### Data management and analysis

Responses were collated by the authors using the survey tool and the results were exported to Microsoft Excel (Microsoft Corporation, Redmond, WA, USA) for analysis.

## Results

### Responses

Totals were calculated for the number of respondents from each category surveyed. Owner responses increased from 287 responses in 2005–2011 to 406 in 2011, private veterinary practitioners responses rose from 72 to 74 for the same periods, only one response was received from welfare groups for 2011, whereas 9 had responded for 2005–2010. Seven local authorities responded for 2005–2010 and 8 responded for 2011. Six category 2 plants responded for 2005–2010 and this fell to 4 responses for 2011. No responses were received from abattoirs for 2011, whereas one had responded for the previous period.

### Foal production

Thoroughbred foal births were 7,588 in 2010, 7,550 in 2011 and 7,546 in 2012.

### Owners - members of the ITBA and HSI affiliates

The 406 horse owners that responded to the survey kept 3,708 horses in 2011. 1,458 (39.3%) were registered or unregistered sporthorses, 1557(42.%) were thoroughbreds 72 (2%) were unregistered thoroughbreds and 621 (16.7%) were cobs, ponies, other types or donkeys. Of those horses for which further information was provided, more than 715 were males, more than 2,040 were females and 792 were geldings; two respondents kept >100 males and >100 females; 840 were <1 year old, 1,362 were 1 to 5 years old, 1,002 were 6 to 10 years old, 554 were 11 to 15 years old and 450 were >15 years old. One respondent kept >100 horses in each of the above age groups (Table 1).

**Table 1:** Total horses kept and number of horses sold and disposed of, arranged by category of sale and disposal 2010 and 2011

**Year**	**2010**	**2011**
Horses kept	2,738	3,708
Private sales	134	147
Public auction	406	606
Sold to dealer	98	61
Retired	82	108
Re-homed	116	44
Euthanasia	61	124
Category 2 plant	32	44
Abattoir	30	41

Responses on identification were provided for 3,613 (97.4%) of the 3,708 horses. 313 (8.7%) had passports and no microchip, 44 (1.2%) had microchips but no passports, 3,066 (84.9%) had both passports and microchips, 153 (4.2%) had neither passports nor microchips and 37 (1%) responded “don’t know”.

386 of the owners (95%) responded to the question on private sales – 38 said yes (9.8%) and 348 (90.2%) said no. The 38 positive respondents sold 147 horses privately, of which 63 (42.8%) were males, 65 (44.2%) were females and 19 (12.9%) were geldings. 12 (8.2%) were < 1 year old, 108 (73.5%) were 1–5 years old, 20 (13.6%) were 6 to 10 years old and 7 (4.7%) were > than 10 years old.

292 of the 406 owners (71.9%) did not sell at public auction but 81 (20%) did so, and they sold 606 horses of which 302 (49.8%) were males 250 (41.3%) were females and 54 (8.9%) were geldings. 202 (33.3%) were < 1 year old, 345 (56.9%) were 1–5 years old, 27 (4.5%) were 6 to 10 years old and 32 (5.3%) were > than 10 years old.

376 of the 406 owners (92.6%) responded to the question on sales to horse dealers. 344 (84.7%) had not sold to dealers and 32 (7.8%) had done so. They sold a total of 61 horses to dealers of which 39 (63.9%) were registered or unregistered sporthorses, 13 (21.3%) were registered thoroughbreds and 9 (14.8%) were cobs ponies or other types.

308 (75.9%) of the 406 owners had not retired any of their horses but 67 (16.5%) had done so. They sent a total of 108 horses into retirement of which 59 (54.6%) were registered thoroughbreds, 32 (30.2%) were registered sporthorses and 17 (15.2%) were cobs ponies or other types. 31 (28.7%) were 5 to 10 years old, 12 (11.1%) were 11 to 15 years old, 42 (38.9%) were 16 to 20 years old and 23 (21.3%) were > 20 years old. Of those 106 horses for which gender information was provided 71 (67%) were females, 12 (9.4%) were males and 23 (21.7%) were geldings. 47 (57.7%) of the 71 females were registered thoroughbreds.

345 (85%) of the 406 owners did not re-home any of their horses but 27 (6.7%) did so. They re-homed a total of 44 horses of which 17 (38.6%) were registered or unregistered sporthorses, 20 (45.5%) were thoroughbreds and 7 (15.9%) were cobs or ponies. 6 (13.6%) were males, 20 (45.5%) were females and 17 (38.6%) were geldings. 5 were <1 year old, 21 were 1 to 5 years old, 9 were 6 to 10 years old, 6 were 11–15 years old and 3 were >15 years old.

311 (76.6%) of the 406 owners did not euthanise any horses but 60 (14.8%) did so. They euthanised a total of 124 horses of which 83 (66.9%) were registered or unregistered thoroughbreds, 27 (21.8%) were registered or unregistered sporthorses and 14 (11.3%) were cobs, ponies or other types including one donkey. Of those 117 horses for which gender information was provided, 20 (17%) were males, 77 (66%) were females and 20 (17%) were geldings. 22 (17.7%) were < 1 year old, 29 (23.4%) were 1 to 5 years old, 16 (12.9%) were 6 to 10 years old, 17 (13.7%) were 11 to 15 years old and 40 (32.3%) were > 15 years old. A rationale for euthanasia was provided on 119 of these 124 horses. 51 (42.9%) were euthanised for medical reasons, 36 (30.2%) were euthanised because of old age and 32 (26.9%) were euthanised for non-medical reasons.

337 (83%) of the 406 owners did not send horses to Category 2 plants, but 32 (7.9%) did so. They sent a total of 44 horses to these plants of which 21 (47.7%) were registered or unregistered sporthorses, 19 (43.2%) were registered or unregistered thoroughbreds and 4 (9%) were ponies. Gender data was provided on 34 of these horses, 6 (17.6%) were males, 26 (76.5%) were females and 2 (5.9%) were geldings. 3 (6.8%) were <1 year old, 10 (22.7%) were 1 to 5 years old, 6 (13.6%) were 6 to 10 years old, 7 (15.9%) were 11 to 15 years old and 18 (40.9%) were >15 years old.

357 (87.9%) of the 406 owners did not send horses to abattoirs but 19 (4.7%) did so. They sent 41 horses to abattoirs of which 34 (82.9%) were thoroughbreds and 7 (17%) were registered or unregistered sporthorses. gender data was provided on 39 (95%) of these horses, of which 32 (82%) were females and 7 (18%) were geldings. Age data was provided on 32 of these horses, 6 (18.8%) were 1 to 5 years old, 5 (15.6%) were 5 to 10 years old, 6 (18.8%) were 11 to 15 years old and 15 (46.8%) were >15 years old.

### Practicing veterinary profession

72 practitioners reported that they had euthanised 197 horses of which 66 (33.5%) were registered or unregistered sporthorses, 86 (43.7%) were registered or unregistered thoroughbreds and 45 (22.8%) were cobs, ponies or donkeys. 23 of these 72 (31.9%) practitioners reported that 18 (78.3%) of their responses related to the euthanasia of these horses at the request of the owner and that 5 (21.7%) were not. Gender data was provided on 96 (48.7%) of these horses, 17 (17.7%) were males, 51 (53.1%) were females and 28 (29.2%) were geldings. Age data was provided on 122 (61.9%) of these horses, 27 (22.1%) were < 1 year old, 18 (14.8%) were 1 to 5 years old, 22 (18%) were 5 to 10 years old, 22 (18%) were 10 to 15 years old and 33 (27%) were >15 years old. Reasons for euthanasia were provided for 116 (58.9%) of these horses, 55 (47.4%) were euthanised on medical grounds, 35 (30.2%) because of old age and 26 (22.4%) for non-medical reasons. Identification data was provided on 104 (52.8%) of these horses, 15 (14.4%) had passports, 48 (46.2%) had passports and microchips, 38 (36.5%) had neither passports nor microchips and 3 (2.9%) were “don’t know” responses (Table 2).

**Table 2:** Number of horses euthanised by the practicing veterinary profession on medical grounds, or at the request of owners, the Gardai or Local Authorities in 2010 and 2011

	**2010**	**2011**
Vets responding	79	72
Total euthanised	237	197
Medical conditions	209	55
Non medical / owner request / old age	13	61
Gardai request	15	1

51 practitioners responded to whether they had been asked to attend abandoned or neglected horses by the Gardai, 10 of them had done so and 41 had not, 3 had done so once, 2 had done so 4 times, 1 had done so 3 times, 1 had done so 6 times and 1 had done so 10 times. These visits involved a single horse 12 times, 2 to 4 horses 14 times and 5 to 10 horses, twice.

One practitioner reported a single euthanasia at the request of the Gardai and this was a 6–10 year old sporthorse that had neither a passport nor microchip.

### Welfare groups

One welfare group responded to the online survey with 2011 data. It reported 11 to 30 calls about abandoned, neglected or roaming horses. 7 calls involved a single horse, 10 involved 2 to 4 horses and 3 related to 5 to 10 horses. This group reported age data on 44 horses, 5 (11.4%) horses were < 1 year old, 19 (43.2%) were 1 to 5 years old, 15 (34%) were 6 to 10 years old and 5 (11.4%) were 11 to 15 years old.

They provided identification data on 56 horses, all 56 (100%) of which had neither passports nor microchips. They reported that they found dead, 4 cobs and 2 ponies and that they had not disposed of any horses through the category 2 plants or abattoirs.

The on-site visits to five welfare societies provided information on acreage available for grazing, staff levels and number of calls they received (Table 3). They ranged from 20 to 160 acres with permanent staff ranging from 3 to 60 and the number of calls ranged from 345, to 3,650. They kept between 16 and 600 equidae on site and fostered between 8 and 400 off-site (Table 4). The breed distribution was 119 non-thoroughbreds and 31 thoroughbreds. The 73 ponies were the preponderant type (Table 5) and although some kept more males than females, others kept more females than males (Table 6). Emaciation, parasitism and lameness were the principal conditions identified on admission (Table 7).

**Table 3:** Welfare Societies – acreage available for grazing, staff (permanent, temporary & voluntary and number of calls received re, unwanted horses)

**Organisation**	**Acreage**	**Staff**			**Number of calls**
	**For grazing**	**Permanent**	**Temporary**	**Voluntary**	
A	20	25 (P&T)		0	345
B	63	2	4	3	ND*
C	56	3	0	Occasional	ND*
D	25	5	0	0	1458
E	160	60	10	10	3650

*ND - not documented.

**Table 4:** Welfare Societies–number of horses, ponies and / or donkeys kept on site or fostered to other sites

**Organisation**	**Numbers**	
	**On site**	**Fostered**
A	16	-
B	64	20
C	38	-
D	20	8
E	600	400

**Table 5:** Welfare Societies–breed and types kept

**Organisation**	**Breed**							
	**TB**	**SH**	**Foals**	**Cob**	**Pony**	**Trotters**	**Irish draft**	**Donkey**
A	1				7			8
B	16	x		x	x			
C	6	14	7	16	27		4	
D	8			2	39	10		15
E								100%

**Table 6:** Welfare societies – gender distribution

	**Genders**				
	**Male**				**Female**
		**Stallions**	**Colts**	**Gelding**	
A		10	30		40
B	30%				70%
C		1	20	12	41
D	70%			3	30%
E		70%			30%

**Table 7:** Welfare Societies – Most common medical problems on relinquishment

**Organisation**	**Most common medical problems on relinquishment**
A	● Leg injuries
	● Skin disease
	● Impalement injuries
	● Lacerating wounds
B	● Severe emaciation
	● Liver disease
	● Problems with feet (laminitis and abscesses most commonly)
	● Parasitic infestations
	● Dental problems
C	● Liver disease
	● Parasite infestations
	● Laminitis
	● Eye injuries
	● Ringworm
	● Emaciation
D	● Emaciation
	● Parasite infestation
	● Lameness
	● Foot issues
E	● Foot problems
	● Lameness
	● Parasite infestation
	● Laminitis

### Local authorities

Eight local authorities responded with 2011 data.

### Calls

The number of calls about abandoned or neglected horses ranged from 5 to 90. 78 of these calls related to one horse, 45 to 2–4 horses, 14 to 5–10 horses, 2 to 11–15 horses, 2 to 16–25 horses, and one each to 26–50 horses and the other to 51–100 horses. There were no reported calls relating to registered or unregistered thoroughbreds or to registered sporthorses.

Breed information was provided on 355 horses, 160 (45%) calls related to unregistered sporthorses and 195 (55%) to cobs ponies and other types. Gender data was provided on 300 of these horses, 56 (18.7%) were males, 173 (57.7%) were females and 71 (23.7%) were geldings.

Age data was provided on 369 of these horses, 28 were < 1 year old, 200 were 1 to 5 years old, 122 were 6 to 10 years old, 5 were 11 to 15 years old and 14 were >15 years old.

### Visits

Six of the eight local authorities reported on the number of visits they made to abandoned or neglected horses. One made 6 visits, one made 18 visits, one made 19 visits and 2 made 40 visits. 28 visits involved one horse, 27 visits involved 2 to 4 horses, 12 visits involved 5 to 10 horses 2 visits involved 15 to 25 horses and one each involved 16 to 50 horses and the other was to 51 to 100 horses.

Breed information was provided on 265 horses, 180 (67.9%) unregistered sporthorses and 85 (32%) ponies.

Gender data was provided on 264 horses; 49 (18.6%) were males, 144 (54.5%) were females and 71 (26.98%) were geldings.

Identification data was provided on 285 horses, 26 (9.1%) had passports, 10 (3.5%) had passports and microchips, 163 (57.2%) had neither passports nor microchips and 86 (30.2%) were categorized as “don’t know”.

### Seizures

Five of the local authorities reported that they had seized a total of 258 horses, one had seized 3 horses, one had seized 14 horses, one had seized 58 horses, one had seized 80 horses and another had seized 103 horses.

Breed information was provided on 132 horses, 30 (22.7%) were unregistered sporthorses and 102 (77.3%) were cobs, ponies, donkeys and other.

Gender data was provided on 97 horses, 30 (30.9%) were males, 57 (58.9%) were females and 10 (10.3%) were geldings.

Identification data was provided on 132 horses, 30 (22.7%) were unregistered sporthorses, 15 (11.4%) were cobs and 69 (52.3%) were ponies, 6 (4.5%) were other breeds, 12 (9.1%) were donkeys and all 132 (100%) had neither passports nor microchips.

### Horses released following seizure

Four local authorities reported release of horses following seizure, two reported release of 2 horses, another released 3 horses and one released 37 horses. Gender data was available on 5 horses, 2 were males and 3 were females. Age data was reported on 5 horses, 1 was <1 year old, 3 were 1 to 5 years old and 1 was 6 to 10 years old.

### Horses found dead or requiring immediate Euthanasia

Two local authorities each reported two horses that were found dead or required immediate euthanasia.

### Horses Euthanised by local authorities

3 local authorities reported a total of 118 horses that they sent to euthanasia, one sent 13 another 40 and a third sent 65. 53 were classified by breed, 38 (71.7%) were ponies and 15 (28.3%) were cobs. 53 had age data, 5 were < 1 year old and 48 were between 1 and 5 years old.

### Category 2 plants

Four Category 2 plants reported that they had processed a total of 1,103 horse carcasses. They supplied breed data on 142 horses, 11 (7.7%) were registered or unregistered sporthorses, 12 (8.5%) were registered thoroughbreds, 51 (35.9%) were cobs, and 68 (47.9%) were ponies. Gender data was provided on 143 horses, 110 were males and 133 were females. Age data was provided on 19 horses, 9 were <5 years old, 4 were 5 to 10 years old, 1 was 11 to 15 years old 1 was 16 to 20 years old and 4 were >20 years old.

The number of horses slaughtered in the three Department approved slaughtering plants in 2012 was 11,402 (Table 8) and the number slaughtered in the two Local Authority approved plants was 12,805 which gives a total horse slaughterings figure of 24,207 for 2012. Central Government Statistics (Table 8) show a decrease of 1,173 in horses slaughtered in DAFM abattoirs from 2011 to 2012 with an increase of 328 in Local Authority seizures and an increase of 531 in those sent by Local Authorities to Category 2 plants.

**Table 8:** Central government statistics

	**2010**	**2011**	**2012**
Number of horses seized under the Control of Horses Act 1996	2,364	2,601	2,929
Number of horses disposed by Local Authorities to Category 2 plants	659	1,592	2,123
Slaughtered at DAFM approved abattoirs	7,296	12,575	11,402
Slaughtered at Local Authority approved plants	2,464	5,233	12,805

Trichinella testing has been carried out for all Irish horse carcasses for human consumption at the Irish Equine Centre since 2005. This data have been used to chart the monthly and annual totals of horses slaughtered at Irish abattoirs (Table 9).

**Table 9:** Monthly totals of horses slaughtered for human consumption at all Irish abattoirs January to December, 2010 to 2012 – based on IEC samples for equine Trichinella testing data

**Month**	**2010**	**2011**	**2012**
January	193	974	1956
February	549	1338	2056
March	424	2077	1953
April	406	1685	1404
May	503	1206	1752
June	483	1483	1647
July	537	1082	1547
August	736	669	1735
September	1,252	1810	1894
October	1,550	1442	3056
November	1,270	2093	3322
December	653	707	1731
**TOTAL**	8,556	16,566	24,053

## Discussion and conclusion

The data from the ITBA and the affiliates of HSI show a similar pattern for the current study period as in the period that preceded it. They represent responsible owners whose horses are identified and who dispose of them in an appropriate manner. It is reasonable to assume that only a small number of owners of similar high value horses, who may or may not even be members of these associations, behave irresponsibly.

As before, the majority of horses euthanised by private veterinary practitioners are euthanised on medical grounds, because of old age or for non-medical reasons. The profession retains its extensive involvement in the care of unwanted horses through its responses to welfare groups and local authorities.

Welfare societies provide an important service but they have limited resources. There is a need for welfare groups to form some association, to reduce competition between them with all the media demands that that can entail, and by doing so, to pool their resources to the benefit of all.

Unwanted horses continue to be a significant draw on public finances because of the demands they place on the local authorities, in terms of calls, visits, seizures, releases and dispatch to category 2 plants.

The vast majority of unwanted horses in Ireland are disposed of through the Category 2 plants and the abattoirs. There has been a progressive increase in disposal through both of these systems throughout the economic downturn and this trend continued upward throughout 2011 and 2012.

There is an ongoing need for these very important, efficient and humane means of disposal and that their absence or closure as in the USA, on foot of the current horsemeat scandals, would rapidly create major welfare and social issues.

The Irish Equine Centre Trichinella data corroborates the DAFF abattoir totals and it usefully includes submissions from non-DAFF Abattoirs. This data is readily available on a monthly basis and it provides the single most useful index unwanted horses in Ireland.

This data showed clearly that then present crisis had not begun to resolve. However, at the time of submission of this report for publication (July 2013), the January to June 2013 Irish Equine Centre Trichinella sample total of 6,562 was a fall of from 10,768 i.e. a reduction of 35% on the figure for the same period in 2012. The well publicised fraudulent labeling of horsemeat as beef was identified in January and February 2013. Two of the slaughter houses in operation at that time took commercial decisions to cease slaughtering horses and to concentrate on the slaughter and processing of other meats for which they had larger markets and higher returns. These commercial decisions were not in any way related to the identification of mislabelled beef in other Irish processing plants and no such inference can be drawn.

## Competing interests

The authors do declare that they have no competing interests.

## Authors’ contributions

DL initiated the study, obtained the funding, provided the basic format of the study, wrote the first draft of the manuscript and acted as study team leader. VD participated in the study design and supervised RJ and D’OT who worked on the survey and data from and visits to the welfare groups, respectively. DL and VD worked together on the subsequent drafts and on the final draft. All authors read and approved the final manuscript.
